# Tinkering With Testing: Understanding How Museum Program Design Advances Engineering Learning Opportunities for Children

**DOI:** 10.3389/fpsyg.2021.689425

**Published:** 2021-07-08

**Authors:** Maria Marcus, Diana I. Acosta, Pirko Tõugu, David H. Uttal, Catherine A. Haden

**Affiliations:** ^1^Department of Psychology, Roosevelt University, Chicago, IL, United States; ^2^Department of Psychology, Loyola University Chicago, Chicago, IL, United States; ^3^Institute of Psychology, University of Tartu, Tartu, Estonia; ^4^Department of Psychology, School of Education and Social Policy, Northwestern University, Evanston, IL, United States

**Keywords:** parent-child interactions, engineering practices, reflection, learning, museums, informal education

## Abstract

Using a design-based research approach, we studied ways to advance opportunities for children and families to engage in engineering design practices in an informal educational setting. 213 families with 5–11-year-old children were observed as they visited a tinkering exhibit at a children’s museum during one of three iterations of a program posing an engineering design challenge. Children’s narrative reflections about their experience were recorded immediately after tinkering. Across iterations of the program, changes to the exhibit design and facilitation provided by museum staff corresponded to increased families’ engagement in key engineering practices. In the latter two cycles of the program, families engaged in the most testing, and in turn, redesigning. Further, in the latter cycles, the more children engaged in testing and retesting during tinkering, the more their narratives contained engineering-related content. The results advance understanding and the evidence base for educational practices that can promote engineering learning opportunities for children.

## Tinkering With Testing: Understanding How Museum Program Design Advances Engineering Learning Opportunities for Children

Advancing engineering learning opportunities for children is a national priority in the United States as part of an effort to increase the quantity, quality, and diversity of the pool of future engineers and other STEM professionals (National Academy of Engineering [NAE], and National Research Council [NRC], 2009). The *Framework for K-12 Science Education* (National Research Council [NRC], 2012), *Next Generation Science Standards* (NGSS Lead States, 2013), and policy reports on the topic (e.g., National Research Council [NRC], 2009; National Science Board [NSB], 2010; National Academies of Sciences, Engineering, and Medicine [NAS], 2018) emphasize the potential for STEM-related experiences in early childhood to pay big dividends in terms of advancing skills that prepare pathways to future STEM educational opportunities and careers. For example, children who spend time in STEM-related museum exhibits tend to show more interest in STEM, do better in STEM-related classes, show better scientific reasoning abilities, and express more interest in STEM subjects and careers (National Research Council [NRC], 2009, 2012, 2015). Studies of autobiographical memory stories of career scientists also support the notion that informal learning experiences in early childhood advance skills that can open doors to future science and engineering pursuits (e.g., Maltese and Tai, 2010; Jones et al., 2011; Crowley et al., 2015). Nevertheless, to realize these potential benefits of early STEM experiences, we need to understand how to design and facilitate experiences in early childhood that can deepen engagement in disciplinary practices of science and engineering. Our work aims to grow the empirical base for educational practices that support young children’s engagement in engineering design during informal learning experiences in museums.

Our work focuses on *tinkering*—a form of playful, open-ended problem-solving involving real tools and materials (Vossoughi and Bevan, 2014). Museums and other informal learning institutions have increasingly integrated design-make-play experiences such as tinkering into STEM-relevant offerings for children and families. With this move, however, has come the realization among educators and researchers (e.g., Honey and Kanter, 2013; Bevan, 2017; Pagano et al., 2020) that not all tinkering activities engender children’s engagement in engineering design practices as outlined in the *Next Generation Science Standards* (NGSS Lead States, 2013) and the *Framework for K-12 Science Education* (National Research Council [NRC], 2012). In particular, NGSS and the *Framework* break the engineering design process into three stages: (1) defining an engineering problem, (2) creating and testing possible solutions, and (3) improving the design solution. Whereas the expectations around these big ideas become more complex across K-12, even for the youngest learners, the NGSS and *Framework* place strong emphasis on the development and testing of solutions and iterative refinement. Although tinkering and engineering are not identical (Martinez and Stager, 2013), when young children playfully explore a problem space and test and iteratively adjust their creations during tinkering, this engagement in disciplinary practices may especially benefit learning about engineering (e.g., Berland et al., 2013; Petrich et al., 2013; Vossoughi and Bevan, 2014).

### Approach

As we see it, tinkering is nearly an ideal context for exploring ways to support informal engineering learning opportunities. Our approach marries constructivist ideas about the importance of learning through direct experiences interacting with objects (e.g., Piaget, 1970) with sociocultural theories emphasizing that learning is co-constructed through socially shared and scaffolded (guided) activities with others (e.g., Vygotsky, 1978; Hirsh-Pasek et al., 2003; Rogoff, 2003; Gaskins, 2008). The emphasis in museums on hands-on activities with objects to promote learning reflects Piaget’s (1970) view that representations of knowledge emerge from and are tied to actions on objects (see also Bruner, 1996). More recently, work on embodied cognition underscores the importance of physical actions for learning (Martin and Schwartz, 2005; Martin, 2009; Macedonia, 2014; Pouw et al., 2014). Nonetheless, children’s engagement in museum exhibits and programs is frequently social, and consistent with sociocultural theories that the social milieu can provide critical mechanisms for learning from hands-on activities. Children’s work with objects often becomes the focus of social-communicative exchanges between children and caregivers that can support understanding of underlying ideas and learning for a number of reasons (e.g., Narayanan and Hegarty, 2000; Jant et al., 2014). For example, parent-child interactions during tinkering can provide mechanisms for making physical engagement with objects a focus of explicit learning. Moreover, parent-child social-communicative exchanges can also facilitate the process of what Sigel (1993) called *distancing* and what Goldstone and Sakamoto (2003) called concreteness fading—learning to focus less on the objects and more on the concepts and knowledge that can be gained from object manipulation.

To contribute to both theoretical and practical understandings of ways to support engineering learning we employ design-based research (DBR), a form of use-inspired basic research (e.g., Barab and Squire, 2004; Joseph, 2004; Sandoval and Bell, 2004). In visitor studies, educational psychology, and other applied fields, it is often unclear how to integrate results gained through basic experimental research methods into practice (and vice versa). DBR seeks to bridge this gap between basic research and application. In Stokes’ (1997) four quadrants of scientific research addressing understanding (yes/no) and use (yes/no), DBR falls in Pasteur’s quadrant (yes/yes), named for the scientist whose renowned scientific discoveries had immediate use in stopping bacterial contamination (pasteurization) and preventing diseases (vaccines). DBR aims to advance both theoretical understandings and practical applications. Importantly, to meet the challenges of DBR, our university researcher-children’s museum practitioner partnership is fully collaborative (Allen and Gutwill, 2016; Haden et al., 2016). The decision-making power is shared in all aspects of the work, from the identification of a problem that could advance theory and practice, to the design of tinkering programs, iteration of practices, and ways of assessing learning (Haden, 2020).

DBR involves multiple phases—cycles—within one study. A cycle begins with the theory-driven design of practices, and encompasses analysis of the impacts of those practices, with the outcomes of each cycle serving as inputs to the redesign of practices and theory refinements in the next cycle. Through successive iterations, and improvements in theoretical and design ideas, one should expect that educational practices improve in terms of advancing learning (Joseph, 2004). Our DBR focused on a specific problem of practice: whereas open-ended, tool-focused programs in a tinkering exhibit in a children’s museum (e.g., Woodshop Plus) engendered tool use and joint engagement by parents and children, there was little evidence of deep engagement in engineering (Pagano et al., 2020). We advanced the idea that tinkering programs offering a function-focused problem-solving goal—in this case, to make something that rolls—would increase engagement in engineering practices, and in particular, children’s testing of their creations. Given that testing is a key aspect of the engineering design process (NGSS Lead States, 2013), we thought that if we could encourage testing, it would foster children’s engagement with and learning about engineering practices.

### Tinkering With Testing

Research shows that young children are eminently capable of engaging in nascent engineering and science practices once thought beyond their years (e.g., Gelman and Brenneman, 2004; National Research Council [NRC], 2009; Lachapelle and Cunningham, 2014; Lucas and Hanson, 2014; English and Moore, 2018). Moreover, tinkering activities can provide an important entry point for participation in specific disciplinary practices of engineering emphasized in engineering education (National Research Council [NRC], 2009, 2012; NGSS Lead States, 2013). Nonetheless, to bring engineering learning to fruition through tinkering, children need to not only make something through exploration of tools and materials, but also participate in the *engineering design process* of creating, testing, and enhancing and improving their solutions to problems. We studied iterative cycles of a program that posed an engineering design problem: make something that rolls. Engineering design problems are characterized by *criteria* and *constraints*. In the Make it Roll program, the criteria for success were specified—the creation needed to roll, not slide. The constraints included the materials that were available in the exhibit that could be used to make wheels and axles, e.g., plastic bottle caps, drinking straws. Wheels and axles (used to reduce friction) are one of six types of simple machines that engineers use on a daily basis to solve problems. Determining whether and how to make the wheels or axles spin was a primary focus of the testing and redesigning we aimed to observe among families.

Our approach to supporting engagement in the engineering design process was twofold, involving exhibit design and facilitation strategies. In the first DBR cycle in this study—Make it Roll I—we created exhibit spaces for testing, including small ramps at the worktables and a large ramp. The design challenge “Make Something that Rolls” was written on the chalkboard, and facilitation staff stated the challenge verbally when they greeted visitors entering Tinkering Lab exhibit. In the subsequent two cycles, Make it Roll II and Make it Roll III, the design iterations and facilitation strategies changed to increase children’s engagement in testing their tinkering creations. There were alterations to the location and design of the large ramp (Figure 2), as well as iterations of a *facilitated orientation* for visitors as they entered the exhibit (Figure 3). During the orientation, museum staff introduced key engineering information about wheels and axles (e.g., “For your car to roll, either the axle needs to spin or the wheels need to spin freely.”) and encouraged testing of different model cars (e.g., “Go ahead and test the car on the ramp to see if it rolls.”).

**FIGURE 1:**
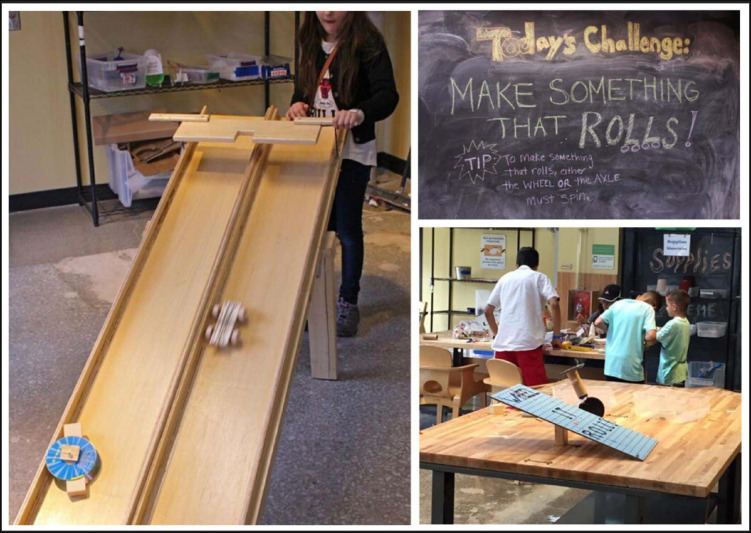
Tinkering Lab exhibit during the first cycle of the Make it Roll program.

**FIGURE 2:**
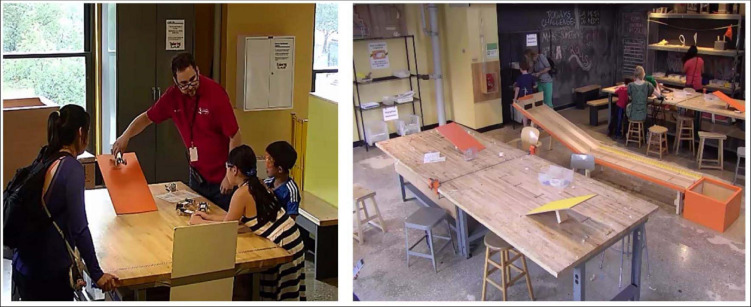
Programming space and Tinkering Lab exhibit during the second cycle of the Make it Roll program.

**FIGURE 3:**
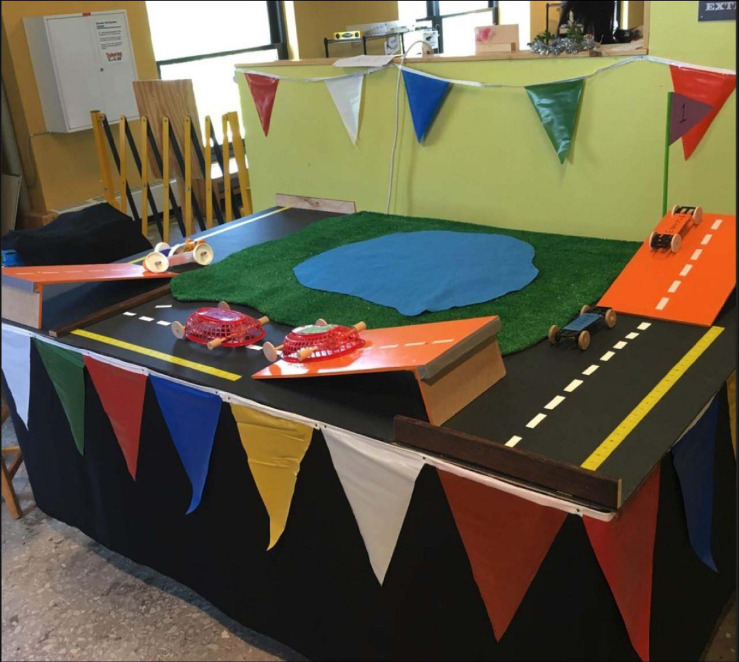
Programming space during the third cycle of the Make it Roll program.

The decision to introduce the facilitated orientations in the second cycle was guided by prior work showing that when families were offered engineering related information at the outset of their museum experience, it benefits children’s engagement in an engineering activity, and their recall of science and engineering information weeks later (Benjamin et al., 2010; Haden et al., 2014). However, whereas in prior work researchers primed families with exhibit-related information (van Schijndel et al., 2010; Jant et al., 2014; Eberbach and Crowley, 2017; Willard et al., 2019), these interventions rarely simulated the kinds of interactions families might have with museum staff. We were interested in how opportunities to engage with practices of engineering through exhibit design and staff facilitation might affect children’s engagement in testing during tinkering and engineering learning.

### Children’s Narrative Reflections About Learning

We also assessed what children may have learned from their tinkering experiences in a way that would be organic to the museum setting (Callanan, 2012; Acosta et al., 2021). After children finished tinkering, we invited them to respond to a series of open-ended prompts to tell a short narrative about their tinkering project. Most of the prior work on using narratives as a measure of children’s learning from exhibit experiences has involved parent-child reminiscing (Benjamin et al., 2010; Haden et al., 2014; Jant et al., 2014; Pagano et al., 2019, 2020). Nevertheless, by the age of five, children are able to tell reasonably coherent stories about recently experienced events in response to fairly open-ended prompts (Fivush et al., 1995; Reese et al., 2011). Moreover, although there has been more attention given to family conversations during exhibit experiences as mechanisms for scaffolding STEM learning in museums (e.g., Crowley et al., 2001a,b; Leinhardt et al., 2002; National Research Council [NRC], 2009; Sobel and Jipson, 2016; Callanan et al., 2020), what children say about their experiences shortly afterward can be viewed both as an extension of the learning process and an outcome of learning (Acosta et al., 2021). With respect to children’s narrative reflections as an assessment of learning outcomes, the content of children’s reflections can offer insights into what children understood about their experiences. Further, in the context of our work, analysis of the content of children’s reflections can address whether and to what extent our tinkering interventions support children’s engineering learning through tinkering.

### Hypotheses

Across three DBR cycles, we observed parent-child pairs who visited the Tinkering Lab exhibit at a children’s museum during one of the three programs. Children’s narrative reflections were elicited immediately after tinkering. We advanced the following hypotheses:

1. If our efforts to iterate the Make it Roll program were successful at increasing engagement in engineering practices, families in the later cycles of our DBR would engage in more testing and redesigning than those in the initial cycle.
2. Families’ engagement in testing would be positively related to children’s talk about engineering in their narrative reflections immediately after tinkering. We also predicted that the more children and parents engaged in testing, the less children would talk about tools and materials in their post-tinkering narrative reflections.

## Methods

### Participants

The sample consisted of 213 families with 5–11-year-old children. We recruited families as they entered the *Tinkering Lab* exhibit at the Chicago Children’s Museum. Sixty-four families visited during the first cycle of the Make it Roll program in Summer 2016; 83 families visited during the second cycle of the Make it Roll program in Summer 2017; and 66 families visited during the third cycle of the Make it Roll program in Summer 2019. The analytic sample reflects only the families who made something that rolled during their visit to *Tinkering Lab*, 59, 80, and 66 families, in cycles 1, 2, and 3, respectively. Table 1 provides demographic information by DBR cycle.

**TABLE 1:** Demographic information for families in the three cycles of the Make it Roll program.

	**Make it Roll**		
	**Cycle 1**	**Cycle 2**	**Cycle 3**
	*N* = 59	*N* = 80	*N* = 66
Age of target child in years [Mean(SD)]	7.45 (0.82)	7.09 (0.83)	7.18 (1.01)
Sex of target child (#)			
Female	30	33	34
Male	29	46	32
Not reported	0	1	0
Race/Ethnicity of target child (%)			
White	67.8	42.5	57.6
African American/Black	3.4	6.3	9.1
Asian	3.4	6.3	6.1
Hispanic/Latino	22.0	16.3	13.6
American Indian/Alaska Native/Native Americans	0	0	1.5
Mixed	3.4	15.0	4.5
Other	0	1.3	1.5
Not reported	0	12.5	6.1
Education of target parent (%)			
Completed some high school	1.7	1.3	1.5
High school graduate	5.1	6.3	1.5
Associate degree	17.0	13.8	16.7
Bachelor’s degree	27.1	25.0	30.3
Completed some postgraduate	3.4	6.3	3.0
Master’s degree	23.7	33.8	33.3
Ph.D., Law, Medical Degree	10.2	11.3	6.1
Not reported	11.9	2.5	7.6

### Procedure

The study procedures were approved under Loyola University Chicago IRB protocol #1776, *Advancing Early STEM Learning Opportunities through Tinkering and Reflection.* The study took place in the *Tinkering Lab* exhibit at Chicago Children’s Museum. *Tinkering Lab* is a workshop space that is equipped with a range of tools and repurposed materials, which during the Make it Roll cycles included tools (e.g., hole punchers, scissors, tape, and glue) and materials (straws, sticks, CD disks, spools, bottle caps, cardboard, wood dowels, skewers, paper food trays, and other recyclables) that could be used to make something that rolls. With written informed consent from parents and children’s assent, we audio and video recorded individual families as they tinkered. Families picked which one adult and one child in the family group would wear the microphones and would be the targets for the observation. Families were encouraged to interact as they normally would and could stay in the exhibit for as long as they wanted.

#### Design-Based Cycles

Families who participated in this study came to *Tinkering Lab* during one of three cycles of our design-based research (DBR) focused on the Make it Roll program. The cycles varied regarding the design of the exhibit and the information that was provided to families by facilitation staff members:

##### Cycle 1

During the first cycle of the Make it Roll program, museum staff greeted families as they entered the exhibit, invited them to make something that rolls, and pointed out the available tools and materials. They also assisted with tool use (e.g., hot glue gun, saw) and answered any questions visitors had. As illustrated in Figure 1, on each of the tables in the large workshop area, there were small tabletop ramps with fun encouragements written on the top (e.g., “Rock and Roll It”). In the far corner of the exhibit there was a large six foot wooden ramp.

##### Cycle 2

During the second cycle of the Make it Roll program, museum staff offered families a brief *facilitated orientation* as they entered the workshop through a smaller programming space. As shown in Figure 2, the programming space was set up with one station featuring a tabletop ramp and various model vehicles, some with rotating wheels and axles, and some with stationary wheels. The models were made of materials that were not available in the large workshop space where the families would make their own creations. Using the ramp and models, museum staff provided information about wheels and axles (“If something is to roll, either the wheels move by themselves or the wheels move with the axle.”), encouraged testing (e.g., “Go ahead and test the car on the ramp to see if it rolls.”), and identified differences between sliding and rolling (e.g., “Why did this car slide and that one roll?”). These orientations were unscripted, and staff were encouraged to use their natural speaking style, although they received training on the information that should be included in the orientation about wheels and axles, testing, and sliding vs. rolling. As in Cycle 1, staff provided support for tool use as families tinkered.

The design of the workshop space was also iterated. Some materials to make wheels in the first cycle (CD disks) were removed because they proved to make poor wheels, and others were made more available, specifically a greater variety of plastic caps of various sizes. As shown in Figure 2, the tabletop ramps were redesigned to look like a roadway, and the large ramp was placed in the center of the room. The large 6-foot ramp was also made more colorful, and the incline less steep, to make it more difficult for contraptions to slide instead of roll down the ramp. There was also a six foot straightaway added to the end of the ramp, along which a measuring tape that offered information about distance traveled from the bottom of the ramp toward a catch bin at the end (see Figure 2).

##### Cycle 3

During the third cycle of the Make it Roll program, museum staff continued to provide facilitated orientations to families before they entered the exhibit, but we iterated the presentation from what was offered in Cycle 2. As shown in Figure 3, families were presented with the challenge to make something that rolls and then invited to explore three stations, each containing a small ramp and various models. At the first station, one model did not include an axle and had the wheels glued on the sides, while the second model had an axle that spun freely to rotate the wheels. At the second station, one model had different sized pairs of wheels, whereas the second model had wheels that were the same size on the front and back. At the third station, one model had wheels positioned too high on the body of the vehicle so they could not touch the ground, and the other had wheels that touched the ground. Facilitators encouraged parents and children to compare and test each pair of models to determine how they were different and which one rolled. Again, facilitators received training, but did not have a set script. Regarding the design of the large workshop space, there were no changes from Cycle 2; the tabletop ramps and the large ramp were the same and their positions were the same in the space. Immediately after the facilitated orientation, families were invited to enter the exhibit and make something that rolls.

#### Children’s Narrative Reflections

Immediately after tinkering, 50 children in cycle 1, 30 children in cycle 2, and 63 children in cycle 3 were engaged in a narrative reflection task by a researcher. The reduced sample sizes in cycles 1 and 3 are due to either children electing not to complete the narrative reflection, the family needing to leave after tinkering, or technical difficulties. In cycle 2, as part of our larger project, our data collection split the sample to collect either parent-child reminiscing data (see Pagano et al., 2020) or children’s narrative reflections immediately after tinkering.

**FIGURE 4:**
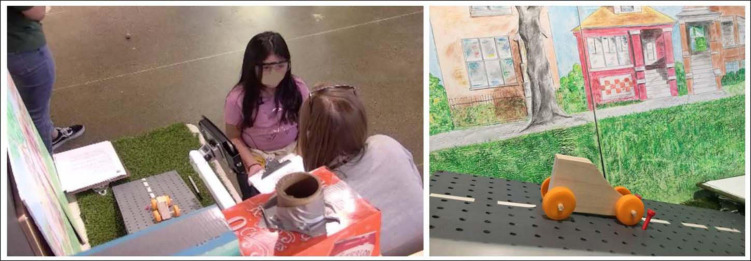
Narrative reflection station.

The narrative reflection began by inviting the children to place their creation on a ramp against a colorful backdrop and then use a tablet computer to take a picture of their creation (see Figure 4). The researcher then elicited the children’s reflections using the following open-ended prompts: (1) What did you do in Tinkering Lab today? (2) How did you do it? (3) What did you learn today? Given our design-based approach, we also iterated these post-tinkering reflections. Specifically, we added the following questions in cycle 2: “Did somebody help you? Tell me how you worked together.” “Did you test your creation? Did it roll?” In cycle 3, we began the interview with questions about the orientation: (1) “When you entered the Tinkering Lab, did you explore the test tracks in the small workshop? What did you do there?” (2) “Was it helpful? How was it helpful?” (3) “Did you learn anything from comparing the creations? Tell me all about it.” We then asked the children about their tinkering experiences. Although these questions were worded slightly differently than in the previous iterations, they were covering the same topics. (4) What did you do in the large workshop in Tinkering Lab? (5) How did you make it? Tell me all about making it. (6) Did you try it out, did you test it? What happened when you tried it out? (7) Did you have to fix your creation? Why did you have to fix it? How did you fix it? Tell me all about it. (8) Did somebody help you? Tell me how you worked together. (9) What did you learn today? (10) Anything else you would like to tell me about making your creation roll? All of the questions were also followed with general prompts (e.g., “Anything else you would like to share?” “Tell me more.”) to elicit more information from the children. These reflections were video and audio recorded.

### Coding

The interactions during tinkering were scored directly from the video records. Children’s post-tinkering reflections were coded from verbatim transcripts. The procedures for establishing inter-rater reliability were the same for all coding systems. Specifically, two researchers independently coded 20% of the records and compared their results. Once reliability was established, no single reliability estimate in any cycle was below Cohen’s kappa (κ) 0.70.

#### Engagement in Engineering Design Practices

To capture engagement in engineering practices, we focused on those instances when either the child or the parent physically tested to see if their creations or parts of their creations rolled. A *test* was scored when the child or parent attempted to spin the wheels or axles of their creation on a ramp, or while holding it on or above the worktable. We distinguished tests from test repetitions. A *test repetition* was scored when the child or parent tested the creation without making any changes to the creation between tests. Kappas averaged 0.85 for children and 0.90 for parents. We further coded what happened after each test or test repetition performed by the child or the parent as either: (1) redesigning –making changes to the creation (e.g., repositioned a wheel to make it spin or touch the ground, swapped the drinking straw for a wooden stick to serve as an axle), (2) decorating—adding non-functional parts to the body of the creation (e.g., added a flag, wrote their name on the creation), or (3) testing was taken over by the partner—after the test by the parent or the child the other partner took the creation and conducted a test (e.g., the child was testing the creation and the parent took the creation and conducted another test without doing anything to the creation in between these tests), (4) other/undefined. Kappas for these subcodes averaged 0.83 for children, and 0.86 for parents.

#### Children’s Narrative Reflections

The transcripts of the children’s narrative reflections were coded using a system developed by Acosta et al. (2021). The coding unit was *instance of occurrence*; any word or group of words that fit the coding category were coded, except repetitions. The content of the children’s talk during the narrative reflections was coded when it involved (1) naming or describing *tools and materials* (e.g., “We used the tape so the wheel could stick on.” “I used the black straws.”), and (2) *engineering-related talk*, including talk about testing, redesigning, and how the wheels and axles help make something roll (e.g., “We tested on the ramp.” “I learned that my wheels need to spin for my car to roll”), predictions and explanations (e.g., “Well it’s not very good at racing because it goes sideways.”), and mathematics reflecting progress toward the engineering goal (e.g., “My car rolled to the two foot measure.”). The kappas averaged 0.89.

## Results

### Preliminary Analyses

Initial analyses addressed whether children in the three DBR cycles were equivalent in terms of child age and gender. As shown in Table 1, there were no significant age differences, [*F*_(__2, 204)_ = 2.86, *p* = 0.059, η^2^ = 0.03], nor significant gender differences across the three cycles, χ^2^(2, *N* = 204) = 1.52, *p* = 0.47, Cramer’s *V* = 0.09.

### Engagement in Engineering Design Practices During Tinkering by DBR Cycle

When looking across DBR cycles, 93% of the children tested their creation at least once, and the percentage of children who tested their creation at least once did not vary by cycle, χ^2^(2, *N* = 205) = 4.84, *p* = 0.089, Cramer’s *V* = 0.15 (Cycle 1: 88.1%; Cycle 2: 97.5%; Cycle 3: 90.9%). Children worked on their creations with a parent, and 81.5% of the parents tested the creation at least once. In the later cycles (Cycle 2: 81%; Cycle 3: 91%), a higher percentage of parents tested the creation compared to the initial cycle (71%), χ^2^(2, *N* = 205) = 8.03, *p* = 0.018, Cramer’s *V* = 0.20. In more than half of the families, the first test was conducted by the child, with no difference across cycles in the percentage of families where this was the case (Cycle 1: 61.1%; Cycle 2: 61.3%; Cycle 3: 51.6%), χ^2^(2, *N* = 196) = 1.60, *p* = 0.449, Cramer’s *V* = 0.09. When examining what happened after children’s first test, we also found no differences across cycles in the percentage of children who continued to work on their creation to improve or redesign it (Cycle 1: 56%; Cycle 2: 54%; Cycle 3: 65%), χ^2^(12, *N* = 192) = 16.23, *p* = 0.181, Cramer’s *V* = 0.21.

We hypothesized that families in the later cycles of the program would engage in more testing and redesigning than families in the initial cycle. Therefore, we examined whether families’ engagement in testing varied based on the DBR cycle (Make it Roll 1, 2, or 3) they participated in. As shown in Table 2, compared to children in the first cycle, children in the second and third cycles conducted significantly more tests [*F*_(__2, 204)_ = 8.83, *p* < 0.001, η^2^ = 0.08], and test repetitions [*F*_(__2, 205)_ = 6.88, *p* < 0.01, η^2^ = 0.06]. Likewise for parents, those in the second and third cycles conducted significantly more tests than parents in the first cycle [*F*_(__2, 204__)_ = 10.17, *p* < 0.001, η^2^ = 0.09]. Parents did not perform many test repetitions, but there were more test repetitions by parents who participated in Cycle 2 than Cycle 1 [*F*_(__2, 204)_ = 6.57, *p* < 0.01, η^2^ = 0.06].

**TABLE 2:** Families’ engagement in testing by DBR cycle.

	**Make it Roll**					
	**Cycle 1**		**Cycle 2**		**Cycle 3**	
	***M***	***SD***	***M***	***SD***	***M***	***SD***
**Children**						
Tests	4.49_a_	4.48	7.15_b_	5.57	8.89_b_	7.18
Test repetitions	0.81_a_	1.71	4.20_b_	6.56	4.63_b_	8.28
**Parents**						
Tests	2.58_a_	2.85	5.55_b_	6.79	7.29_b_	6.64
Test repetitions	0.10_a_	0.48	0.94_b_	1.85	0.62	1.12

*Across individual rows, means with subscript a differ from means with subscript b at p < 0.05 in pairwise tests with Bonferroni adjustment for multiple comparisons.*

Next we considered what happened after children and their parents performed a test or test repetition. Table 3 shows the proportion of tests or test repetitions that were followed by redesigning, retesting, decorating, or the partner taking over. As shown in Table 3, most tests or test repetitions were followed by redesigning. However, contrary to our hypothesis, we did not find that redesigning increased across DBR cycles, for children [*F*_(__2, 189)_ = 0.41, *p* = 0.66, η^2^ = 0.00], or parents [*F*_(__2, 166)_ = 1.81, *p* = 0.166, η^2^ = 0.02]. Essentially, the iterative improvements in the program increased testing, which in turn engendered further engagement in the engineering process during tinkering. There were fewer tests followed by decorating in Cycles 2 and 3 compared with Cycle 1 for children [*F*_(__2, 189)_ = 7.34, *p* < 0.01, η^2^ = 0.07], and parents [*F*_(__2, 166)_ = 5.29, *p* < 0.01, η^2^ = 0.06].

**TABLE 3:** Proportion of tests followed by redesigning, decoration, and partner taking over.

	**Make it Roll**					
	**Cycle 1**		**Cycle 2**		**Cycle 3**	
	***M***	***SD***	***M***	***SD***	***M***	***SD***
**Children**						
Redesigning	0.36	0.36	0.37	0.25	0.41	0.28
Decoration	0.11_a_	0.22	0.02_b_	0.07	0.04_b_	0.11
Parent takes over	0.05	0.12	0.05	0.10	0.08	0.13
**Parents**						
Redesigning	0.60	0.35	0.58	0.30	0.49	0.29
Decoration	0.13_a_	0.30	0.01_b_	0.04	0.05	0.17
Child takes over	0.10	0.17	0.18	0.27	0.21	0.23

*Across individual rows, means with subscript a differ from means with subscript b at p < 0.05 in pairwise tests with Bonferroni adjustment for multiple comparisons. The proportions do not add up to 1, because of other/unclassifiable behaviors that occurred infrequently after a test.*

### Linking Engineering Engagement During Tinkering and Children’s Narrative Reflections

We hypothesized that the more families tested during tinkering the more their children would talk about engineering in the post-tinkering narrative reflections. Further, we thought that the more testing the parents and children engaged in during tinkering the less the children would talk about tools and materials in their narrative reflections. Recall that we iterated these post-tinkering reflections and the number of questions asked varied across cycles. Therefore, we calculated the proportion of children’s talk about materials and tools and about engineering-related talk, dividing the frequency of each of these codes by the total number of questions asked. As shown in Table 4, overall, children’s tests during tinkering were positively associated with their talk about engineering in the post-tinkering narrative reflections. As also predicted, parents’ test and tests repetitions during tinkering negatively correlated with children’s talk about tools and materials in their reflections. In other words, the more engineering was the focus of the tinkering activity the less “tool talk” in the children’s reflections. What is more, when we looked at the correlations by DBR cycle, in Cycles 2 and 3, we saw significant positive correlations between children’s tests during tinkering and their engineering talk in the reflections, and in Cycle 3, positive correlations between children’s test repetitions during tinkering and engineering talk in the reflections. For parents, the negative associations between test repetitions during tinkering and children’s talk about tools and materials in the reflections were only statistically significant for Cycle 2. Overall, this pattern of results suggests that when testing by children and their parents during tinkering is more frequent, as was the case in Cycles 2 and 3, children’s reflections included more talk about engineering, and less talk about tools and materials.

**TABLE 4:** Partial correlations between families’ tests and children’s reflections.

	**Engineering during tinkering**			
	**Children’s**	**Children’s test**	**Parents’**	**Parents’ test**
	**tests**	**repetitions**	**tests**	**repetitions**
**Content of children’s narrative** **reflections across cycles**				
Discussing materials and tools	–0.15	−0.20*	−0.27**	−0.20*
Engineering-related talk	0.31**	0.16^*t*^	0.12	–0.04
**Cycle 1**				
Discussing materials and tools	0.14	0.05	–0.03	0.11
Engineering-related talk	0.15	0.15	0.20	0.08
**Cycle 2**				
Discussing materials and tools	0.15	–0.02	−0.35^*t*^	−0.38*
Engineering-related talk	0.44*	0.01	0.25	0.00
**Cycle 3**				
Discussing materials and tools	–0.09	–0.11	0.04	–0.08
Engineering-related talk	0.34**	0.28*	0.02	–0.15

**p < 0.01, *p < 0.05, ^t^p < 0.10.

## Discussion

Using a design-based research approach, we studied ways of enhancing engineering learning opportunities for children in an informal educational setting. Taken together, the results suggest exhibit design and facilitation strategies that can promote children’s engagement with authentic practices of engineering during tinkering, specifically, testing and redesigning. The work also illustrates how design-based research methods can help us understand and support learning in real-world contexts.

### Engagement in Engineering Design Practices

Our results demonstrate that children can and do participate in the engineering design process during tinkering by creating, testing, and re-designing. This was true in all three cycles of our “function-focused” tinkering program that posed a specific engineering challenge to make something that rolled, and included exhibit design features to support testing and iterating toward a functional goal. Prior work suggests that parents and children talk more about engineering during such function-focused tinkering programs than tool-focused programs (Pagano et al., 2020), and here we show too that hands-on engagement during the Make it Roll program was engineering-rich. The majority of the children we observed tested to see if their creation did indeed roll. After testing their creation, more than half of the children continued to work on their creation to redesign or improve it. Although relatively speaking the proportion of tests that were followed by redesigning did not increase across cycles, the number of tests did, meaning that by encouraging testing we also encouraged further engagement in the engineering design process. Nonetheless, just as not all tinkering is engineering (Martinez and Stager, 2013), adding a place to test ones design during a tinkering activity does not in and of itself maximize the potential for engineering learning through tinkering. Indeed, in contrast to the first version of the program, the second and third iterations paired the design feature of a ramp with facilitation strategies by museum staff members. It was the later versions of the program that led families in this study to engage in the most testing, and in turn, redesigning, which are key engineering practices (National Research Council [NRC], 2012; NGSS Lead States, 2013).

We focused on testing and redesigning because they move *making* something to *engineering* something. Authentic engagement in an engineering design process does not stop with the first rendering of a design, nor with the first test. Rather, engineers use testing to gather information about how effective, efficient, durable, etc. their design is, to compare different design solutions, and determine what works best to solve the problem within the given constraints (National Research Council [NRC], 2012). Nonetheless, the practice of testing might not yield the best possible solution to a problem unless the ensuing redesign features the application of relevant engineering principles. This idea provided the motivation for the introduction of the facilitated orientations by museum staff members, Cycle 2, and refinements of this strategy in Cycle 3, to highlight key engineering information about wheels and axles to families at the outset of the tinkering challenge.

In this project, we focused on ways that museum staff could provide relevant information to families. Prior work suggested that this might be successful. For example, Haden et al. (2014) carried out an intervention in a building construction exhibit wherein some visitors received information about triangular bracing before creating their own skyscrapers. Haden et al.’s project and some others (Eberbach and Crowley, 2017; Marcus et al., 2018; Willard et al., 2019) offering key information to visitors to support learning were fashioned to mimic museum programming. However, it is rare for empirical work to involve museum staff in carrying out the interventions (Franse et al., 2021). The current study exemplifies how this approach can be especially fruitful, yielding ecologically valid tests of effectiveness of practices that are directly applicable to enhance informal learning opportunities.

### Children’s Narrative Reflections

Our work also involved an effort to connect hands-on engagement during tinkering with an assessment of what children might have learned from their experiences. We elicited children’s narrative reflections immediately after tinkering. We found that particularly in the second and third cycles of our design-based research, the more children engaged in testing and retesting during tinkering, the more their narratives contained engineering-related content. The following example from a child who participated in Cycle 3 illustrates this result. Here the child describes several tests, and how they led to diagnosing what might need to be fixed and trying different solutions, in a series of tests and redesigning efforts:

Child: The first time I tested it, it was all, it started going wonky.

Researcher: Oh no!

Child: And then the second time we tested it, we realized it’s the back wheels, so then we changed it.

Child: The third time we tested it, we added a couple of more things to make it more even like light in the back.

Child: And then the final time I tested it, it worked!

Researcher: Wow and did it roll?

Child: Yes.

Researcher: It did?

Researcher: Very cool.

Child: But it didn’t roll that far.

Researcher: That’s okay.

Child: It went to one feet.

A challenge with assessing learning in museum environments is to do so in ways that respect the character of an informal educational setting. Our museum partners previously developed a special multi-media exhibit—*Story Hub: The Mini Movie Memory Maker—*to encourage families to tell stories together about their exhibit experiences. In fact, as part of our larger project, a subset of the families we observed in this project in Cycle 2 were invited to reflect together on their tinkering experience in Story Hub (see Pagano et al., 2020). We developed the procedures used in this study to elicit children’s independent reports of their learning (in contrast to family reminiscing), in part, because earlier work suggested that children will report more engineering content when they have their projects with them than when they do not (Pagano et al., 2019). We also wanted to create a simple procedure that could be put into practice by museum staff, one that could potentially further boost learning from hands-on experiences by virtue of the opportunity for children to verbally express their experiences.

Reflection is foundational in modern STEM education. Part of the reason for this is the ways that reflection can reveal learning outcomes. Indeed, in our work, the content of the children’s reflections were diagnostic of how changes in the design of the Make it Roll program were advancing engineering learning opportunities. As children’s engagement in the engineering practice of testing increased across variations of the tinkering program, so too did children’s talk about engineering in their reflections. As parents tested more across iterations of the program, children talked less about tools and materials. The content of the reflections therefore provided insights into what the children understood about their experiences, and potentially what was most meaningful and memorable, information that is useful to not only researchers, but also educators and parents, who seek to support children’s STEM learning.

Narrative reflections also present the opportunity to extend children’s learning beyond the hands-on experience itself and may help with consolidation of learning from hands-on activities such as tinkering (Marcus et al., 2018; Pagano et al., 2019). Reflection can extend the initial learning through hands-on activity to support the creation of a richer and more meaningful representation of the experience, one that may be more memorable and transferable beyond the museum’s walls (Haden, 2014; Marcus et al., 2017). In support of this idea, Marcus et al. (2021) had some families reflect on a building experience in a museum exhibit immediately afterward, whereas others did not engage in the post-building reflection. They found that compared to families who did not engage in the narrative reflection at the museum, those who did talked more about STEM when working on a related building activity at home. In light of this and other similar work (e.g., Jant et al., 2014), success in increasing the engineering talk in the children’s reflections can be important as part of an overall process of learning and learning transfer that a museum visit may engender.

### Limitations and Future Directions

This research makes important contributions to the literature on children’s learning as well as to informal educational practices. Nevertheless, there are several limitations of the work. First, our design-based research involved successive iterations of the tinkering program which were introduced one after another into the exhibit space, and therefore random assignment of participants was not possible. Relatedly, our sample sizes for each cycle varied based on the duration of the program in the museum’s calendar, and the days it was possible to collect data. During Cycle 2, on different days of the week, post-tinkering narrative reflections were either collected in *Story Hub* or elicited from the children by a researcher—the post-tinkering narrative reflections by children that are presented in this paper. The uneven number of participants across cycles is not ideal. Additionally, although museum staff received training on the information that was to be included in the orientations regarding wheels and axles, testing, and sliding vs. rolling, there was variation in how this information was delivered to families. Again, this is an example of how our work differed from a standard experimental study. Nonetheless, it would be interesting in future work to consider how natural variations in the ways the staff delivered the orientations—such as to what extent they directly explained or engaged families in a give and take conversation to convey the information—might further predict variation in the families’ subsequent engagement in tinkering, and the children’s narrative reflections.

This project did not combine a focus on hands-on testing and the conversations parents and children engaged in together during tinkering. This is an important next step, as indeed, the way we frame our larger project theoretically speaking is that conversations add layers of understanding to children’s experience beyond hands-on activity alone (Haden et al., 2016). Moreover, it is clear from research on STEM learning in museums that parent-child conversations can support children’s learning. In future work, we are especially interested in examining contingencies between verbal and non-verbal behavior during tinkering. For example, when a child engages in testing, what does the parent say? Does the parent ask a question or provide an explanation? This approach is encouraged by recent work by Callanan et al. (2020) that shows that the timing of parents’ talk when children are engaging in a hands-on activity can provide a specific mechanism by which joint hands-on and conversational engagement scaffolds children’s STEM learning.

### Implications for Enhancing Engineering Learning Opportunities for Children in Informal Educational Settings

Our work is situated at a children’s museum and also grounded in a unique partnership between university researchers and museum practitioners. Engaging in developmental psychology research in museums is a growing trend, but the nature of the working relationships forged between researchers and museum practitioners is highly variable (Callanan, 2012; Sobel and Jipson, 2016; Haden, 2020). One critical dimension along which these working relationships vary is the degree to which the research might offer insights into effective practices for supporting children’s learning. Working *with* the museum, we iterated the Make it Roll program to determine how to maximize this potential engineering learning opportunity. Important indicators of our success came in the form of our observations of parents and children testing their creations, and children’s narrative reports of what they learned from the tinkering experience. Our results point to several specific practices that can be readily implemented in museums. One is providing families with exhibit-related information to support their engagement in science and engineering practices. Another is offering places for testing to encourage participation in engineering practices. After Cycle 1, the ramp was redesigned, not only to encourage measurement of distance, but also to lessen success due to the creation simply sliding. In other words, the design of the testing station was altered to require use of key engineering principles—to make the wheels or axles rotate. Our measure of testing is one that can be observed live, so that museum educators could alter their testing stations to promote engagement with the engineering concepts relevant to the task at hand. Finally, we also see the narrative reflection procedure as a tool that educators can use to understand what works to promote learning in their spaces.

The opportunity to engage in jointly negotiated collaborative research with the children’s museum is important not only in advancing our understanding of children’s STEM-related learning. The work can also directly impact educational practices that can support that learning. This is an important effort broadly speaking, because so much STEM learning happens outside of school, with estimates of children spending 80% of their waking hours learning in informal educational environments, including museums (National Research Council [NRC], 2009). Research-practice partnerships like the one our team enjoys can provide critical insights into children’s learning in real-world contexts, while at the same time advancing practices that enhance STEM learning opportunities for children.

## Data Availability Statement

The raw data supporting the conclusions of this article will be made available by the authors, without undue reservation.

## Ethics Statement

The studies involving human participants were reviewed and approved by the Institutional Review Board, Loyola University Chicago. Written informed consent to participate in this study was provided by the participants’ legal guardian/next of kin. Verbal assent was also obtained from children. Consent was obtained from the individual(s), or minor(s)’ legal guardian/next of kin, for the publication of any potentially identifiable images or data included in this article.

## Author Contributions

DU and CH were PIs for the NSF grant that supported this work. MM, DU, and CH contributed to conceptualization and study design. MM, DA, and PT contributed to the data collection. MM, DA, PT, and CH designed and executed the coding. MM conducted the statistical analyses. MM and CH wrote the first draft of the manuscript. All authors contributed to manuscript revision, read, and approved the submitted version.

## Conflict of Interest

The authors declare that the research was conducted in the absence of any commercial or financial relationships that could be construed as a potential conflict of interest.
